# The Doctor’s Self-Inflicted Wrongs

**Published:** 1902-08

**Authors:** 


### TEXAS MEDICAL JOURNAL,

AUSTIN, TEXAS.

A MONTHLY JOURNAL OF MEDICINE AND SURGERY.

EDITED AND PUBLISHED BY
F. E. DANIEL, M. D.

ASSOCIATE EDITOR:
WITTEN BOOTH RUSS, M. D.,
San Antonio, Texas.

Published Monthly at Austin, Texas. Subscription price $1.00 a year in advance.

Eastern Representative: John Guy Monihan, St. Paul Building, 220 Broadway, New York City.

Official organ of the the West Texas Medical Association, the Houston District Medical Association, the Austin District Medical Society, the Brazos Valley Medical Association, the Galveston County Medical Society, and several others.

### THE DOCTOR'S SELF-INFLICTED WRONGS.

Dr. G. R. Fowler, of Brooklyn, in discussing several claims for professional services rendered which he has succeeded in collecting by due process of law, expresses the hope that the effect of these suits will be to help to put a stop to the impositions practiced upon physicians by those who can, but will not, pay for medical attention.

Medical men have only themselves to blame for impositions of the kind to which Dr. Fowler refers. What are we to expect at the hands of our patients when we constantly do all within our power to convey to their minds the idea that we consider it bad form and altogether improper for a doctor to even present his bill until it is asked for, much less to insist vigorously upon its being paid? Since we allow the laity to regard our claims as of so little importance that they may be paid at "just any time" (which is often taken to mean *never*) it is small wonder that, as a class, we are regarded as poor business men.

It has often been asked why medical men do not put their practice upon a business basis, and, at first thought, it does seem strange, to say the least, that a man with a family, and perhaps himself not more than a "few jumps ahead of the collector," will freely and, to all appearances, gladly extend credit to strangers and known dead-beats, and submit to a loss of more than half of the money actually due him rather than even ask for it at what most men would regard as the proper time. One does not have to seek far, however, for the answer. It is not because the doctor has any especial aversion to money, or because he is willing to wait for it indefinitely, and to risk losing it altogether, nor yet because he thinks it improper or unbecoming to show a "commercial spirit." The *real reason* that he does not attempt to collect his accounts promptly is because he knows that the offices of some of his competitors are always open to receive, without question, all the patients he happens to lose. Experience has taught him that an effort on his part to collect an account in a strictly business-like manner is apt to be followed by the loss of both patient and money.

To make bad matters worse, every community seems to have one or more professional pirates who for the sake of being able to boast of having a large visiting list, or of doing great things in surgery, make every possible effort to attract business from their neighbors. Such men are usually willing to do almost any work for a mere pittance, or even for no fee at all. This they advertise extensively with the laity. And, strange to say, they are regarded by many as being regular and ethical gentlemen. By judiciously managing the "small fry" in the profession their following is, as a rule, both large and enthusiastic. One such *cut-throat* can do more to wreck professional interests in a given community than can any number of advertising quacks or crazy disciples of Mother Eddy, Elisha Dowie and their ilk.

Under the circumstances it is small wonder that medical men cannot charge adequate fees, or collect their accounts as they should, and that even the very tradesmen who present their own claims with frantic haste on the first of every month regard a doctor's bill as little less than a personal insult. R.

